# Borderline Phyllodes Breast Tumors: A Comprehensive Review of Recurrence, Histopathological Characteristics, and Treatment Modalities

**DOI:** 10.3390/curroncol32020066

**Published:** 2025-01-26

**Authors:** Fulvio Borella, Mauro Porpiglia, Niccolò Gallio, Chiara Cito, Lorenzo Boriglione, Giulia Capella, Paola Cassoni, Isabella Castellano

**Affiliations:** 1 Gynecology and Obstetrics 1U, Departments of Surgical Sciences, University of Turin, 10126 Turin, Italylorenzo.boriglione@unito.it (L.B.); 2 Gynecology and Obstetrics 2U, Departments of Surgical Sciences, University of Turin, 10126 Turin, Italy; niccolo.gallio@edu.unito.it; 3 Pathology Unit, Department of Medical Sciences, University of Turin, 10126 Turin, Italy; giulia.capella@unito.it (G.C.); paola.cassoni@unito.it (P.C.); isabella.castellano@unito.it (I.C.)

**Keywords:** borderline phyllodes tumors, breast cancer, treatment, surgical margins, molecular features, phyllodes tumors, recurrence

## Abstract

Phyllodes tumors account for 2–3% of all fibroepithelial breast tumors and less than 1% of all breast cancers. These tumors are categorized into benign, borderline, or malignant based on cellular atypia, mitotic activity, and stromal overgrowth. Surgical excision with clear margins, ideally greater than 1 cm, is the primary treatment for phyllodes tumors to ensure effective local control. Preoperative diagnosis is challenging due to the clinical and radiological similarities between phyllodes tumors and fibroadenomas. The efficacy and role of adjuvant treatments remain subjects of ongoing debate and investigation. Borderline phyllodes tumors exhibit biological characteristics that straddle the line between benign and malignant, presenting significant clinical and surgical management challenges. Given the rarity of this specific subgroup and the ambiguity of the risk of recurrence or progression to malignant phyllodes, this narrative review aims to provide a comprehensive overview of the recurrence risk associated with these tumors.

## 1. Introduction

Phyllodes tumors represent a rare entity, comprising 2–3% of all fibroepithelial breast tumors and less than 1% of all breast cancers [1,2]. Primarily, these tumors affect women aged 35 to 55 and are rare in men [2,3]. Based on cellular atypia, mitotic activity, and stromal overgrowth, phyllodes tumors are categorized as benign, borderline, or malignant [4]. The primary treatment for phyllodes tumors, irrespective of their grade, is surgical excision to achieve clear margins, ideally greater than 1 cm, to ensure effective local control. Both tumor excision and mastectomy can effectively manage low recurrence rates, provided that clear margins are maintained [5]. However, due to the clinical and radiological similarities between phyllodes tumors and fibroadenomas, preoperative diagnosis is often challenging. Consequently, initial surgical enucleation of phyllodes tumors frequently results in inadequate margins [5]. Typically, surgical treatment is sufficient by itself, but there are instances where some medical centers propose the addition of chemotherapy and radiation therapy, such as in cases of recurrent phyllodes tumors following mastectomy or in tumors exhibiting significant stromal overgrowth. However, the role of adjuvant treatments remains a subject of ongoing debate and investigation [5]. Borderline phyllodes tumors display biological features that lie between benign and malignant, posing notable challenges for clinical and surgical intervention. Due to the rarity of this specific subgroup and the ambiguity of the risk of recurrence or progression to malignant phyllodes, this narrative review aims to provide an overview of the management and the recurrence risk associated with these tumors.

## 2. Literature Search

**Figure 1:**
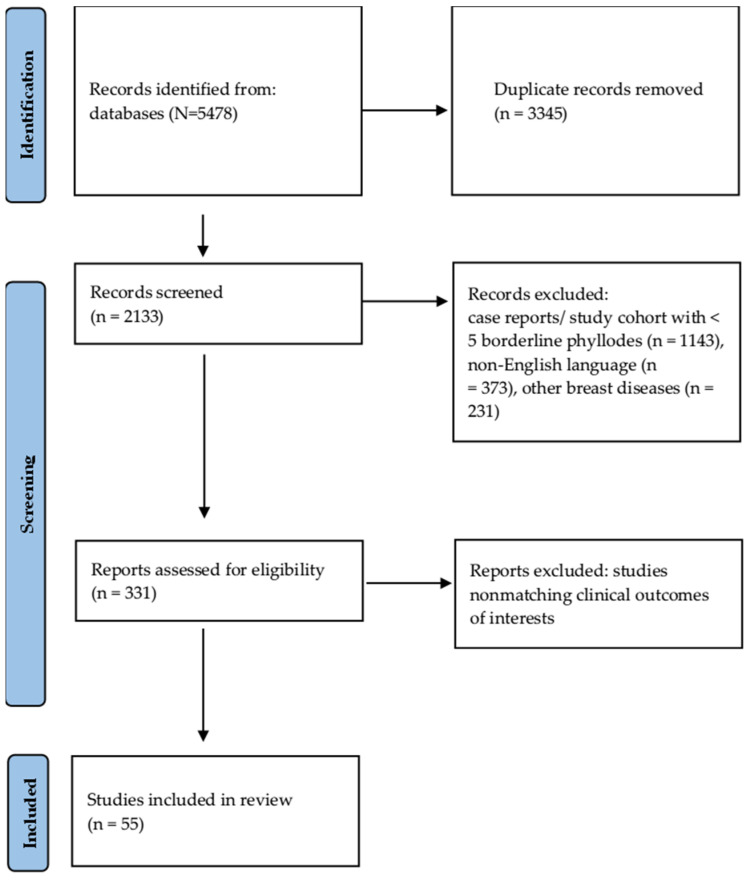
Flowchart of the studies included in the review.

We conducted a literature review of studies published between 1995 and 2024. Our search utilized Medline, PubMed, Scopus, the Cochrane Library, and Web of Science databases. The primary Medical Subject Heading (MeSH) search terms included “breast”, “phyllode”, “phyllodes”, “borderline”, “recurrence”, and “relapse”. Reference lists of the identified articles were searched for other potentially relevant articles. We excluded non-English language papers, case reports, and studies with fewer than five patients. Being a narrative review, for greater completeness, we have also reported the results of other systematic reviews. All articles’ titles and abstracts reporting outcomes of phyllodes borderline tumors were individually screened and reviewed by two authors (FB, NG). If there was disagreement on inclusion, a consensus was obtained after discussion. The results of the literature search and study selection process are summarized in the flow diagram (Figure 1).

## 3. Risk of Recurrence

Descriptive studies performed between the late 1990s and the first half of the 2000s did not show any particular risk factors in predicting recurrence in borderline phyllodes tumors [6,7,8,9]. In a comprehensive study of phyllodes tumors in Asia, the recurrence rates were 10.9% for benign, 14.4% for borderline, and 29.6% for malignant tumors [10]. In a separate German cohort, the recurrence rates were 8% for benign, 20% for borderline, and 50% for malignant tumors, with distant metastases noted in 9% of patients with malignant tumors. Generally, the literature reports 10–17% recurrence rates for benign, 14–25% for borderline, and 23–30% for malignant phyllodes tumors [10]. Asian patients were observed to have higher recurrence rates compared to non-Asian patients [11]. A systematic review and meta-analysis revealed pooled local recurrence rates of 8% for benign, 13% for borderline, and 18% for malignant phyllodes tumors [12]. The risk of local recurrence was significantly higher when comparing borderline tumors to benign tumors (odds ratio [OR]: 2.00; 95% confidence interval [CI] 1.68–2.38) and malignant tumors to borderline tumors (OR: 1.28; 95% CI 1.05–1.55) [12]. Interestingly, in most studies, no patient with borderline phyllodes developed distant metastases or died of the disease [13,14,15,16,17,18,19,20,21,22].

## 4. Pathological Features

### 4.1. Histology

Macroscopically, phyllodes tumors are firm, well-defined, lobulated masses with slit-like spaces and a slightly granular texture [23]. Occasionally, cyst formation is seen, while hemorrhage is rare [23]. There is significant stromal proliferation microscopically, creating slit-like or leaf-like patterns [23]. The stroma varies in cellularity, from hypo- to hypercellular regions, especially under the epithelium. Stromal cells show varying nuclear pleomorphism, from bland to sarcomatous [23]. Mitotic activity correlates with cellularity. Sometimes, extensive stromal expansion hides the ductal epithelium, known as stromal overgrowth [23]. In 1982, the World Health Organization (WHO) classified phyllodes tumors as benign, borderline, and malignant based on histopathologic characteristics [24]. A benign phyllodes tumor has well-defined borders, mild stromal cellularity, minimal atypia, fewer than five mitotic figures per 10 high-power fields (HPF), and no stromal overgrowth or malignant components [4]. Borderline phyllodes tumors show well-defined or focally permeative borders, moderate stromal cellularity, mild to moderate atypia, no malignant components, and five to nine mitotic figures per 10 HPF [4]. Malignant phyllodes tumors have pronounced stromal cellularity, atypia, permeative margins, stromal overgrowth, and at least 10 mitotic figures per 10 HPFs [6]. Mitosis count, cellular atypia, and stromal cellularity increase with tumor grade. Other features, like high microvessel density, are more common in borderline and malignant tumors [25]. Risk factors for local recurrence include mitotic activity, tumor border type, stromal cellularity and atypia, stromal overgrowth, tumor necrosis, and fibroproliferation [12,26,27]. In a study of 166 patients, aggressive behavior was associated with younger age, larger tumor size, higher mitotic count, marked atypia, stromal overgrowth, hypercellularity, necrosis, and heterologous differentiation (*p* < 0.01) [28]. Cytological atypia was also noted as significant by other authors [29]. The 4-year relapse incidence for benign/borderline tumors was 7%, with only one patient dying from a borderline tumor [28]. Higher tumor-infiltrating lymphocytes were found in borderline and malignant phyllodes but did not relate to survival [28]. Zhou et al. proposed a nomogram to predict recurrence risk based on tumor border, tumor residue, mitotic activity, stromal cell hyperplasia, and atypia [30].

### 4.2. Molecular Markers

Several markers have been associated with higher recurrence rates, including MMP-14, Ki67/MIB-1, Six-1, PAX3, FoxC2, TWIST, CXCR4, VEGF, stromal Yes-associated protein (YAP), cellular E-cadherin, and CD10. Conversely, stains such as EGFR, HER2/neu, membranous E-cadherin, MMPs 1, 2, 7, 9, 11, and 13, and tissue inhibitors of metalloproteinases (TIMP) 1, 2, and 3 were not linked to oncologic outcomes [31,32,33,34]. Conflicting results for CD117 were noted in two studies. Three studies examined the prognostic significance of p53: two indicated an association between p53 staining and disease-free as well as overall survival [32,35], while one study found no significant correlation. Ki67 (MIB-1) is a well-established proliferation marker extensively studied in breast cancer. Its expression levels are routinely assessed to determine tumor aggressiveness and guide therapeutic decisions, especially in differentiating between various subtypes of breast cancer [36,37,38]. Patients with MIB-1 levels over 11.2% had an HR of 5.2 for recurrence-free survival and 5.8 for overall survival. In a cohort study of 118 patients, Niezabitowski et al. found that Ki67 > 11.2% was associated with a relative risk of death of 5.12, effectively categorizing malignant phyllodes tumors into two distinct prognostic groups [35].

In a study of 38 cellular fibroadenomas and phyllodes tumors, including various grades per WHO classification, a panel of immunohistochemical stains (p53, CD117, phospho-Histone3, mdm2, cdk4) and mutational analysis of 26 tumors across 30 cancer-related genes were performed [39]. Higher-grade phyllodes tumors exhibited increased staining for p53 and phospho-Histone3, whereas CD117, mdm2, and cdk4 showed no differential expression across grades. Mutational analysis identified an S8R substitution in FBX4 in three cases (one benign, two borderline), which is more common in phyllodes tumors (11.5%) than other tumors. High cyclin D1 expression was noted in FBX4 S8R cases, though not specific [39].

Several studies have aimed to establish a molecular classification for phyllodes tumors. Comparative genomic hybridization studies reveal recurrent chromosomal imbalances such as +1q, −6q, −13q, −9p, −10p, and +5p. While no chromosomal aberrations have been uniquely identified for phyllodes tumors, Lae et al. reported that low-grade (benign) and high-grade (borderline/malignant) phyllodes tumors form two distinct genetic groups based on genomic alterations [40]. High-grade phyllodes tumors consistently exhibit 1q gain and 13q loss, whereas low-grade phyllodes tumors show few or no alterations. Jones et al. corroborated these findings in their array-CGH analysis of 126 phyllodes tumors [41]. However, Lv et al. observed that 1q gain did not correlate with tumor grades [42], and Lu et al. found that 1q gain predominantly occurred in benign phyllodes tumors, suggesting the need for further research [43]. The loss of 13q in phyllodes tumors indicates a potential role for the RB1 gene in phyllodes tumor oncogenesis or progression [40]. Additionally, frequent deletions of 9p21, associated with loss of p16INK4A protein expression, were noted in borderline/malignant phyllodes tumors [41]. Multiple genes have been linked to the development of phyllodes tumors due to their localization in areas of copy number alterations. Among these, EGFR has been highlighted, with fluorescence in situ hybridization showing amplification in 2% to 16% of cases [44,45]. Furthermore, gene expression and immunohistochemistry studies have indicated the activation of several signaling pathways, including insulin-like growth factor (IGF) and Wnt/β-catenin, in phyllodes tumors. A study employed targeted next-generation sequencing (NGS) to detect somatic mutations in formalin-fixed paraffin-embedded specimens from patients with malignant, borderline, and benign phyllodes tumors. NGS identified mutations in mediator complex subunit 12 (MED12), specifically affecting the G44 hotspot residue, in 67% of cases across all histologic grades. Additionally, loss-of-function mutations in TP53 and detrimental mutations in tumor suppressors RB1 and NF1 were found exclusively in malignant tumors. High-level copy-number alterations, including amplifications in IGF1R and EGFR, were predominantly observed in malignant tumors. This comprehensive analysis elucidates the genomic landscape of phyllodes tumors, highlights molecular markers corresponding to histologic grade, broadens the range of tumors with frequent MED12 mutations, and identifies IGF1R and EGFR as potential therapeutic targets in malignant phyllodes tumors [46]. MED12 mutations were also identified by other researchers [47,48,49,50]. A recurrent clonal hotspot mutation in the TERT promoter (-124 C>T) was observed in 52% and TERT gene amplification in 4% of phyllodes tumors [50]. Sequencing analysis of a cohort of fibroepithelial breast tumors revealed that the frequency of TERT alterations increased from benign (18%) to borderline (57%) and malignant phyllodes tumors (68%; *p* < 0.01), with TERT alterations being associated with higher levels of TERT mRNA (*p* < 0.001) [50]. No TERT alterations were found in fibroadenomas. The authors concluded that TERT alterations might drive the progression of phyllodes tumors and could aid in the differential diagnosis between phyllodes tumors and fibroadenomas [50].

## 5. Surgical Management and Adjuvant Therapy

### 5.1. Mastectomy vs. Conservative Surgery

A cohort study that included 11 borderline phyllodes tumors (of which 8 relapsed with distant metastasis) suggested that mastectomy and adjuvant radiation therapy were associated with better disease control; however, the study included a high number of malignant phyllodes tumors [51]. The role of mastectomy for better local control of malignant and borderline phyllodes tumors was also suggested in another study [52].

Other authors assessed the surgical outcomes of 193 phyllodes tumors, including 33 borderline cases [53]. For patients with borderline phyllodes tumors, the type of surgical procedure was the only factor significantly associated with tumor recurrence. Among the six locally recurrent tumors, two were benign, one was borderline, and three were malignant. One malignant recurrent tumor metastasized to the lung one year after recurrence. Local recurrence was most frequent following local excision (*p* = 0.046).

However, a systematic review showed that for benign and borderline phyllodes tumors, no significant differences in local recurrence risk were found between breast-conserving surgery and mastectomy (benign OR 0.68, 95% CI: 0.12–3.78; borderline OR 1.14; 95% CI: 0.29–4.51). Breast-conserving surgery significantly increased the risk of local recurrence only for malignant phyllodes tumors (OR 2.77; 95% CI: 1.33–5.74) [54]. Similarly, a subgroup analysis of another systematic review and meta-analysis indicated that breast-conserving surgery, compared to mastectomy, and positive surgical margins, compared to negative margins, were significantly associated with increased local recurrence risk only in malignant phyllodes tumors [8].

### 5.2. The Role of Surgical Margins

The National Comprehensive Cancer Network (NCCN) guidelines suggest that excisional biopsy with complete mass removal, without aiming for surgical margins, is sufficient for benign phyllodes tumors. Conversely, for malignant or borderline disease, wide excision with surgical margins greater than 1 cm is recommended [55,56]. But this issue remains controversial.

Recently, a large cohort study including 129 borderline phyllodes out of 439 total focused on the impact of surgical margins on recurrence. Of the 129 borderline phyllodes tumors, 11 (8.5%) experienced local recurrence. However, the authors found that surgical margin was not an independent risk factor (HR: 2.280; *p* = 0.091) [57].

Additionally, a large multicenter Danish study that included 89 borderline phyllodes tumors did not find a correlation between surgical margins and the risk of recurrence [13]. Other authors recommended clear margins only in small phyllodes with a high mitotic count [13]. Despite these results, several studies support the role of surgical margins in the risk of recurrence. A large multicenter study [49] of 362 phyllodes (235 malignant and 127 borderline) showed that for borderline tumors, only positive margins (*p* = 0.044) independently increased the risk of local recurrence. A sub-analysis performed by Choi et al. observed that patients with negative margins < 1 cm, younger age, and larger tumor size are independent risk factors for increased local recurrence [58].

Another study investigated 150 phyllodes tumors (65 malignant and 85 borderline) [59]. Among the borderline tumors, 18 (21.2%) experienced local recurrence, and 2 (2.4%) experienced distant metastasis. A surgical margin of less than 1 cm (HR = 2.567; 95% CI 1.137–5.793, *p* = 0.023) and age under 45 years (HR = 2.079; 95% CI 1.033–4.184, *p* = 0.040) were identified as independent risk factors for local recurrence. Additionally, a surgical margin of less than 1 cm (HR = 3.074; 95% CI 1.622–5.826, *p* = 0.001) and a tumor size greater than 5 cm (HR = 2.719; 95% CI 1.307–5.656, *p* = 0.007) were found to be independent risk factors for disease-free survival [59].

Another study that included 29 out of 117 borderline phyllodes suggests that wide surgical margins should be obtained for this neoplasm for better local control [60]. An additional case series, which included only 3 borderline tumors out of 59, also suggested obtaining wide surgical margins to improve disease-free survival [61]. Other published case series that included borderline phyllodes tumors emphasize the importance of obtaining clear surgical margins [16,19,61,62,63,64,65,66].

A systematic review including 34 articles showed that across all phyllodes tumor grades, a positive margin significantly increased the risk of local recurrence (odds ratio-OR: 3.64; 95% CI: 2.60–5.12). However, there was no significant difference in the risk of local recurrence between margins < 1 cm and ≥1 cm (OR 1.39; 95% CI: 0.67–2.92) [67].

Some authors noted that large tumor size is also a factor associated with recurrence, especially for borderline phyllodes tumors [2]. A systematic review and meta-analysis aimed to clarify the optimal surgical management for phyllodes tumors, focusing on the impact of margin status and width on local recurrence rates. Independent searches through multiple databases identified 34 relevant studies with high-quality scores. The meta-analysis found that positive surgical margins significantly increased the risk of local recurrence across all phyllodes tumor grades. At the same time, there was no significant difference in local recurrence risk between margin widths of less than 1 cm and those of 1 cm or more. For benign and borderline phyllodes tumors, local recurrence risks were similar between breast-conserving surgery and mastectomy. However, breast-conserving surgery significantly increased the local recurrence risk for malignant phyllodes tumors. The findings suggest different surgical management strategies based on phyllodes tumor grades, with recommendations for re-excision in borderline and malignant phyllodes tumors with positive margins [68].

A recent study performed an 11-institution contemporary (2007–2017) review of clinical practices of phyllodes breast tumors [69]. This extensive multi-institutional study indicates that the majority of patients with phyllodes tumors are benign and can be effectively managed with breast-conserving surgery, presenting a low overall risk of local recurrence. The margin status or width does not significantly affect this risk. The NCCN guidelines, which recommend a 1-cm margin for all phyllodes tumors, are based on broad principles derived from lower-level evidence. Due to the lack of prospective data and the presence of conflicting results from large retrospective studies, the appropriate margin width for phyllodes tumors remains uncertain.

A revision of the management for benign phyllodes tumors should be considered, as they constitute 70% of all phyllodes and are responsible for 60% of second surgeries, although the authors support current margin recommendations for borderline and malignant phyllodes.

### 5.3. Adjuvant Treatment

The role of radiotherapy has been investigated in a systematic review and meta-analysis on borderline and malignant phyllodes tumors [70]: adjuvant radiotherapy for borderline and malignant phyllodes tumors reduced the local recurrence rate in patients undergoing breast-conserving surgery. However, adjuvant radiotherapy did not demonstrate any impact on overall survival or disease-free survival. Indeed, the authors point out several critical issues: certain confounding factors, such as chemotherapy and endocrine therapy, were not considered due to the unavailability of the original data. These factors might influence the recurrence rate. Some selection bias, particularly the tendency to administer adjuvant radiotherapy to patients with later-stage tumors, was a prevalent issue in most studies (furthermore, most cases were malignant phyllodes). The varying follow-up durations may also limit the interpretation of the results. Lastly, all studies included in our analysis were non-randomized controlled trials, with less convincing results than those from randomized controlled trials. More recently, a study reviewed the treatment outcomes for 340 patients with phyllodes tumors (40 borderline) suggesting to consider radiotherapy in case of close surgical margins (<1 cm) [71]. However, other authors do not support the use of adjuvant treatment regardless of the grade of phyllodes tumors [72].

The most relevant cohort studies, including borderline phyllodes tumors of the breast, are summarized in Table 1.

## 6. Discussion and Conclusions

Borderline phyllodes tumors of the breast present a distinct challenge in clinical management due to their intermediate nature between benignity and malignancy. Local recurrence rates for borderline phyllodes tumors vary significantly across different studies (range 0–33%, Table 1), whereas distant metastases and deaths related to borderline phyllodes are rare and reported only by a few authors (Table 1). Our review has highlighted several key factors associated with recurrences and provides insights into optimal surgical management and the potential role of adjuvant therapies.

Positive surgical margins have been consistently identified as a significant risk factor for local recurrence across all grades of phyllodes tumors. Despite this, there remains no significant difference in local recurrence risk between margins less than 1 cm and those 1 cm or greater. This finding underscores the critical need for clear margins to achieve effective local control. For borderline phyllodes tumors, breast-conserving surgery and mastectomy appear to offer similar local recurrence risks, although breast-conserving surgery has been associated with higher local recurrence rates in malignant phyllodes tumors in some studies. Several histopathological markers associated with recurrence—mitotic activity, tumor border characteristics, stromal cellularity, stromal atypia, stromal overgrowth, and tumor necrosis—have been identified as significant predictors of local recurrence and could be evaluated for the management of borderline phyllodes tumors. Furthermore, recent studies have also pointed out molecular classifications based on genomic alterations. Our findings support the recommendation for re-excision in borderline and malignant phyllodes tumors with positive margins to ensure negative margins are achieved. However, the role of adjuvant radiotherapy remains contentious.

### Conclusions

In conclusion, the management of borderline phyllodes tumors requires a nuanced approach that considers individual patient and tumor characteristics. Clear surgical margins are recommended by guidelines and several studies, and the potential benefits of adjuvant therapies should be weighed against their risks on a case-by-case basis. Continued research is essential to refine these strategies and improve patient outcomes.

## Figures and Tables

**Table 1:** Treatment and survival outcomes of the most relevant case series, including borderline phyllodes tumor of the breast.

Study	Year	Borderline Phyllodes/Entire Cohort	Borderline Phyllodes Local Recurrence	Borderline Phyllodes Distant Recurrence	Surgical Treatment (Borderline Phyllodes)	Adjuvant treatment (Borderline Phyllodes)	Features Related to Recurrence	Dead of Disease (Borderline Phyllodes)
Reinfuss et al. [6]	1996	19/170 (11%)	3 (15%)	0	19 BCS	0	N.A.	0
Zissis et al. [7]	1998	14/84 (16%)	2 (14%)	0	11 BCS, 3 mastectomy	N.A.	N.A.	0
Chaney et al. [8]	2000	12/101 (12%)	0	0	N.A.	N.A.	N.A.	0
Cheng et al. [9]	2006	12/182 (6.5%)	0	0	N.A.	N.A.	N.A.	0
Ben Hassouna [29]	2006	16/79 (20%)	5	0	N.A.	N.A.	N.A.	0
Karim et al. [11]	2009	23/65 (35%)	3 (13%)	0	N.A.	N.A.	N.A.	0
Borhani-Khomani et al. [57]	2016	89/479 (19%)	8 (9%)	0	79 BCS, 4 mastectomy, 6 N.A.	N.A.	N.A.	N.A.
Sevinc et al. [21]	2018	14/122 (11%)	0	0	14 BCS	N.A.	N.A.	0
Wada et al. [18]	2018	21/124 (17%)	6 (28%)	0	19 BCS, 2 mastectomy	N.A.	N.A.	0
Ditsatham et al. [15]	2019	33/188 (18%)	2 (6%)	0	23 BCS, 10 mastectomy	5 RT	N.A.	0
Alkushi et al. [17]	2021	11/45 (24%)	0	0	8 BCS, 2 mastectomy, 1 N.A.	1 RT	N.A.	0
Belkacemi et al. [52]	2008	80/433 (18%)	N.A.	N.A.	N.A.	N.A.	Local excision	N.A.
Kim et al. [53]	2013	33/193 (17%)	6 (18%)	1 (3%)	33 BCS, 1 mastectomy	2 RT	Local excision	N.A.
Sain et al. [51]	2023	11/87 (12%)	N.A.	8 (72%)	N.A.	N.A.	Lumpectomy	N.A.
Sotheran et al. [62]	2005	12/50 (24%)	3 (25%)	0	11 BCS, 1 mastectomy	N.A.	Wide surgical margins	0
Jang et al. [64]	2012	42/164 (25%)	9 (21%)	0	N.A.	0	Positive surgical margins, tumor size	0
Wei et al. [68]	2014	63/192 (32%)	10 (16%)	4 (6%)	N.A.	0	Positive surgical margins, tumor size, age	N.A.
Yom et al. [58]	2015	61/285 (21%)	7 (11%)	0	55 BCS, 6 mastectomy	0	Positive margins in small tumors with high mitotic count	1
Tremblay-LeMay et al. [65]	2017	20/101 (20%)	2 (10%)	0	N.A.	N.A.	Positive surgical margins	0
Choi et al. [13]	2018	127/362 (35%)	23 (18%)	0	112 BCS, 15 mastectomy	2 RT	Positive surgical margins	0
Rodrigues et al. [14]	2018	50/183 (27%)	4 (8%)	0	N.A.	N.A.	Positive surgical margins, infiltrative borders	0
Co et al. [19]	2018	64/465 (14%)	14 (22%)	0	N.A.	N.A.	Positive surgical margins	0
Slodkowska et al. [66]	2018	41/133 (30%)	4 (14%)	0	N.A.	4 RT	Positive surgical margins, myxoid stroma, age < 50 years	0
Spanheimer et al. [16]	2019	39/124 (31%)	N.A.	0	30 BCS, 9 mastectomy	N.A.	Age < 40 years, positive surgical margins	0
Noordman et al. [63]	2020	3/57 (5%)	1 (33%)	0	3 BCS	N.A.	Positive surgical margins	N.A.
Lim et al. [20]	2021	21/150 (14%)	3 (14%)	0	N.A.	N.A.	Positive or ≤1 mm surgical margin	
Su et al. [60]	2024	85/150 (57%)	18 (21%)	2 (2.4%)	65 BCS, 20 mastectomy	1 RT	Close surgical margins, tumor size	
Zhou et al. [30]	2018	184/404 (45%)	26 (14%)	0	171 BCS, 13 mastectomy	5 RT	Tumor border, tumor residue, mitotic activity, degree of stromal cell hyperplasia, and atypia	0
Chng et al. [22]	2018	27/259 (10%)	2 (7%)	0	N.A.	N.A.	Mitoses, stromal overgrowth, positive surgical margins	0
Di Liso et al. [28]	2020	30/166 (18%)	2 (7%)	0	28 BCS, 2 mastectomy	0	Marked cellular atypia, heterologous differentiation	1 *
Yoon et al. [54]	2023	129/439 (29%)	11 (9%)	N.A.	126 BCS, 3 mastectomy	7 RT	Infiltrative border, large size	N.A.

BCS: breast-conserving surgery, N.A.: not available, RT: radiotherapy, * The distant recurrence follows the local recurrence.
